# Classifying transcription factor targets and discovering relevant biological features

**DOI:** 10.1186/1745-6150-3-22

**Published:** 2008-05-30

**Authors:** Dustin T Holloway, Mark Kon, Charles DeLisi

**Affiliations:** 1 Molecular Biology Cell Biology and Biochemistry Department, Boston University, 5 Cummington Street, Boston, USA; 2 Department of Mathematics and Statistics, Boston University, 111 Cummington Street, Boston, USA; 3 Bioinformatics and Systems Biology, Boston University, 44 Cummington Street, Boston, USA

## Abstract

**Background:**

An important goal in post-genomic research is discovering the network of interactions between transcription factors (TFs) and the genes they regulate. We have previously reported the development of a supervised-learning approach to TF target identification, and used it to predict targets of 104 transcription factors in yeast. We now include a new sequence conservation measure, expand our predictions to include 59 new TFs, introduce a web-server, and implement an improved ranking method to reveal the biological features contributing to regulation. The classifiers combine 8 genomic datasets covering a broad range of measurements including sequence conservation, sequence overrepresentation, gene expression, and DNA structural properties.

**Principal Findings:**

(1) Application of the method yields an amplification of information about yeast regulators. The ratio of total targets to previously known targets is greater than 2 for 11 TFs, with several having larger gains: Ash1(4), Ino2(2.6), Yaf1(2.4), and Yap6(2.4).

(2) Many predicted targets for TFs match well with the known biology of their regulators. As a case study we discuss the regulator Swi6, presenting evidence that it may be important in the DNA damage response, and that the previously uncharacterized gene YMR279C plays a role in DNA damage response and perhaps in cell-cycle progression.

(3) A procedure based on recursive-feature-elimination is able to uncover from the large initial data sets those features that best distinguish targets for any TF, providing clues relevant to its biology. An analysis of Swi6 suggests a possible role in lipid metabolism, and more specifically in metabolism of ceramide, a bioactive lipid currently being investigated for anti-cancer properties.

(4) An analysis of global network properties highlights the transcriptional network hubs; the factors which control the most genes and the genes which are bound by the largest set of regulators. Cell-cycle and growth related regulators dominate the former; genes involved in carbon metabolism and energy generation dominate the latter.

**Conclusion:**

Postprocessing of regulatory-classifier results can provide high quality predictions, and feature ranking strategies can deliver insight into the regulatory functions of TFs. Predictions are available at an online web-server, including the full transcriptional network, which can be analyzed using VisAnt network analysis suite.

**Reviewers:**

This article was reviewed by Igor Jouline, Todd Mockler(nominated by Valerian Dolja), and Sandor Pongor.

## Introduction

Many factors influence the regulation of genes and their protein products within the cell. Chromatin condensation, DNA methylation, and histone acetylation/methylation can affect the accessibility of a gene's cis-regulatory sites to trans-acting factors. On the RNA level, mRNA splicing, mRNA editing, microRNA silencing, and RNA degradation can all affect the ability or efficiency of translating mRNA into active protein [1]. Nevertheless, the primary mode of regulatory control is the association of transcription factors with their target binding sites in DNA. These binding sites occur most often in promoter regions, the stretch of DNA upstream of the transcription start site. The string of nucleotides bound by a particular TF is not identical at every recognized site. Instead, the TF distinguishes a flexible motif, or shared pattern of bases [2,3].

Founding work in discovering and representing binding sites involved the use of position specific scoring matrices (PSSMs) [2-5], which represent the frequency of nucleotide bases at each position in a known motif. Many techniques for discovering and predicting binding sites have been reported [6-13], and an evaluation of the state of the art in unsupervised motif-discovery methods is available [14]. Despite their broad usefulness, detection by PSSM is beset by a high rate of false positive predictions. Some TF matrices can produce predictions at a frequency of 1 in every 500 bp [15]. Often, there is not enough information to construct high quality matrices.

To improve the prediction of which promoters are bound (*i.e*., the target promoters), more sophisticated machine learning approaches can be used. Supervised learning schemes begin with more information and seek to generate classification rules based on a user-provided set of positive and negative examples. In our case, a rule can be learned based on provided training data, and it can be applied to the whole genome to predict new targets of a specific TF. Some work has been published on supervised classification schemes for predicting TF binding targets, and we have briefly reviewed a few of these in our previous work [16,17], which focused on developing and applying a support vector machine [18,19] variant to predict transcription factor binding sites in *Saccharomyces cerevisiae*. We now expand that work to include a total of 163 TFs, revise our machine learning strategy to be more robust, and construct and analyze the gene regulatory network in *S. cerevisiae*. All predictions are now available online, including the full transcriptional network, which can be analyzed in the VisAnt browser [20,21].

Genomic datasets have high dimensionality, meaning that for each example gene there may be thousands or tens of thousands of numerical features to describe it. One example is expression microarray data. If the expression of a gene is tested under 200 experimental conditions (*i.e*., 200 microarrays) then the gene is described by a 200 dimensional vector, each element being the expression under a single condition. Many classification algorithms, *e.g.*, *k*-nearest-neighbors or neural networks, may degrade in performance as the number of features becomes large unless selection criteria are used to drastically reduce the size of the feature space. Support vector machines perform well with high dimensional data and have been shown to provide excellent classification accuracy with many genomic datasets (see Methods).

**Figure 1:**
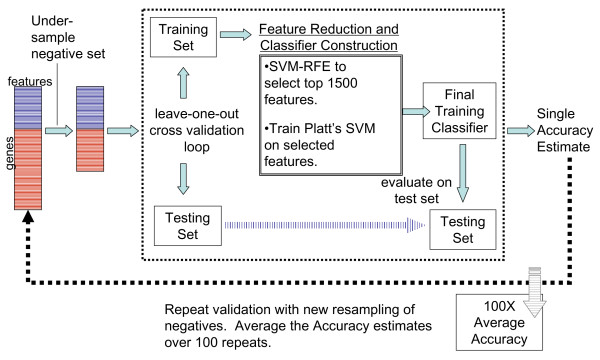
**SVM Framework**. This figure shows the data mining scheme for making TF classifiers. 50 classifiers are constructed for each TF, each using a different random sub-sample of the negative set. First, on the far left, the negative pool for a TF is under-sampled so that it is the same size as the positive set. A classifier built on the training set is evaluated using leave-one-out cross validation (this is shown as a split into train and test sets). For every cross-validation split, the top 1500 features are selected using SVM-RFE (center solid box) and the classifier is trained and finally used to classify the test set. This process is repeated 50 times, and the accuracy for the procedure is the average of the 50 cross-validation accuracies. To classify a potential new target for a TF, all 50 classifiers are applied to the gene's feature vector, and an enrichment score is calculated as discussed in Methods. A score greater than 0.5 indicates a positive classification (an average score greater than 0.95 is used to predict new targets).

We take our positive set from known TF binding sites published in the literature (see Methods) [22-25]. Negatives are a randomly chosen subset of those genes found not to be bound by a TF in ChIP-chip experiments ("not bound " refers to the large group of genes with the highest *p*-values and thus least significant binding). A schematic representation of the classification workflow is presented in Figure 1; a full description of classifier construction and validation is given in Methods.

An SVM classifier is constructed for each TF based on a chosen set of biological features. These features (described in Methods) comprise a diverse set of data including promoter sequence composition, gene expression measurements, phylogenetic conservation of sequence elements, over-representation of promoter sequences, promoter melting temperature, and others. Choosing a negative training set (*i.e*., examples of genes not regulated by the TF) for such a classification is difficult as there is no way to determine which genes are truly *not *bound by the TF. To establish a pool of potential negatives, for each TF we select 600 genes shown as least likely to be bound by ChIP-chip experiments, always assuring that the negative pool is at least three times the size of the positive set. Once a negative pool is chosen, 50 classifiers are constructed for each TF using different random subsamples from the negative pool. Since the negative pool is expected to be noisy, the repeat sampling assures that no single, biased gene group dominates the classifier.

When the negative sets are chosen, they are sampled to be of equal size to the positive sets. This is done since the underlying distributions of positives and negatives are unknown and may vary greatly depending on the TF being studied. Other researchers have also employed such balanced training sets when making classifiers [26]. As discussed below, this has several important consequences for how classifier evaluation measures are interpreted.

Each classifier for a TF is evaluated using a leave-one-out cross-validation (LOOCV) approach, and the reported performance measures (accuracy, and positive predictive value) are the average measurements over all 50 classifiers. Because training sets are balanced an average positive predictive value of 50% is a baseline score which indicates a random classifier. Thus, the average cross-validation measurements are used to determine which TF classifiers can make useful predictions (*i.e*., better than expected by chance).

Post processing is also an important component of transcription factor target prediction. When applied to the entire genome a classifier may work well in finding a set of genes enriched with targets for a regulator, but the set will likely contain too many false positives to be used directly in future analyses. In order to select the best targets, Platt's procedure [27] is used to rank all new predictions. The scores given by Platt's method range from 0 to 1, and are intended to be interpreted (within the training set) as probabilities of any gene being a true positive. Once again, though this is a correct interpretation in the training set, it may result in overly optimistic probability estimates when applied genome-wide. Thus, we use the Platt scores only as a means to rank new predictions, and apply a high cutoff Platt score of 0.95 to identify new predictions for a regulator. Any new prediction must *equal or exceed the 0.95 cutoff on average across all 50 classifiers for a TF*. This restriction will further reduce the number of false positives in our final prediction sets since it is less likely that non-target genes will on the average pass such a high cutoff in 50 classifiers trained with different negative sets. Note here that the 0.95 cutoff is used only for selecting new targets, while the baseline threshold of 0.5 is used when performing LOOCV and evaluating whether a classifier performs better than random. Thus we note that these Platt scores may be interpreted as confidence levels, and that they are correct for selecting targets from balanced datasets used in SVM training and testing. A correction is needed in order to transform these confidence levels to ones which are appropriate to determination of targets from the full genome, in which the number of negatives can outnumber the number of positives by a large factor.

Although we use and report the uncorrected Platt scores here simply as a means to show enrichment for targets we provide, in our Methods section, the calculations necessary to correct these scores to the genomic scale.

Several things set the current work apart from our previous approaches, and allow us to draw biological conclusions that could not otherwise be obtained. Rather than using all features to make a classifier we apply recursive feature elimination to select those that are most relevant. Later the rankings are accumulated across all 50 classifiers (made from resampling the negative set) for a TF to establish a final ranking for each feature. We are thereby able to identify the specific biological attributes which are most useful for separating target genes from non-targets. The simultaneous ranking of all features and identification of those few with dominant relevance allows us to easily discover important biological aspects of regulation. Also, several of our methods have been improved. We introduce a new dataset of *k*-mer counts weighted by their conservation in alignments with sequences from closely related species.

We apply our formalism to the identification of targets for 163 of the ~200 *S. cerevisiae *TFs – these being the regulators with 4 or more established targets – and delineate the repertoire of networks formed by the resulting associations. An analysis of global network properties highlights the transcriptional network hubs; the factors which control the most genes and the genes which are bound by the largest set of regulators. Cell cycle and growth related regulators tend to dominate the former; genes involved in carbon metabolism and energy generation tend to dominate the latter. We use the ubiquitous cell cycle regulator Swi6 as a case study to illustrate the level of insight that can be obtained from a detailed analysis of results. Predictions can be downloaded from a publicly available web server, and the implied networks mined, visualized and analyzed using the VisAnt tool.

## Results and Discussion

### Network Visualization and Analysis

As discussed in Methods below, classifier performance is measured using leave-one-out cross-validation. Since 50 classifiers are trained for each TF each using a different randomly chosen negative set, the reported accuracy is an average over 50 trials. The cross-validation accuracy in training sets for all binding targets (over all classifiers) is 76%. New predictions are the result of averaging the assigned Platt scores from each of the 50 classifiers for a TF and applying a threshold of 0.95.

We note here that the resulting predictions indicate the association of TF and promoter DNA, which is the quantity being measured in ChIP-chip studies. As such, with a binding prediction one cannot necessarily conclude that the bound TF drives expression of the target gene *in vivo*, meaning that the rate of *biological *false positives will need to be determined later in the context of specific experimental conditions.

One of the origins of network structure is combinatorial regulation; *i.e*. single TFs often regulate more than one target, and particular targets are often regulated by more than one TF. Networks can be inferred using a combination of experimental and computational methods and displayed as a repertoire of connections (the cell's network repertoire), different subsets of which are selected by particular environments. The repertoire is available at the VisAnt [20,21] website and can be accessed in the methods table as "TFSVM." Visualization in VisAnt allows predictions to be integrated with many other large scale genomic datasets including protein-protein interaction, gene expression, GO functional annotation, and genetic interaction. VisAnt also allows adjustment of the network based on a user-defined likelihood threshold for accepting an association. Thus predictions can be embedded in networks mined at any specified degree of stringency for further analysis.

The full set of predictions for 163 TFs in *S. cerevisiae *are available our webserver [28]. Users may query either a transcription factor, returning the list of predicted targets, or a gene, returning a list of possible regulators. The Platt scores may also be set by the user, providing an adjustable threshold on the predictions for each TF. In addition, the cross-validated accuracies for all classifiers have been posted online, as well as the top 50 ranked features for each TF-classifier.

### Global Network Properties

In any regulatory network it is of interest to know which genes are most heavily under transcriptional control, and which TFs exert the most control by regulating large numbers of genes. When analyzing global network properties, we limit our analysis to high quality predictions by including only TFs that have a leave-one-out cross-validated classification accuracy greater than 0.6 (there are 130 such TFs), and targets that have a Platt score ≥ 0.95.

The resulting network includes a large number of genes that are under strong regulatory control. In particular 125 genes are regulated by 12 or more transcription factors. These genes show statistical enrichment (*p *≤ 0.05) in several GO biological process categories. The enriched categories are mainly involved in carbon metabolism and energy generation (see Additional File 1), which are crucial functions expected to be under tight control. Other important processes include DNA damage checkpoint, DNA recombination, DNA damage response, acetyl-CoA catabolism, NADP(H) metabolism, and telomere maintenance. With respect to breadth of control, 13 TFs regulate more than 300 genes (known + predicted) each at high Platt score cutoffs (*P *≥ 0.95). This set of TFs includes the pervasive regulators Abf1 and Reb1, as well as TFs involved in the cell cycle, growth, and the stress response (see Additional File 2).

### Local Structure: Swi6 Case Study

**Figure 2:**
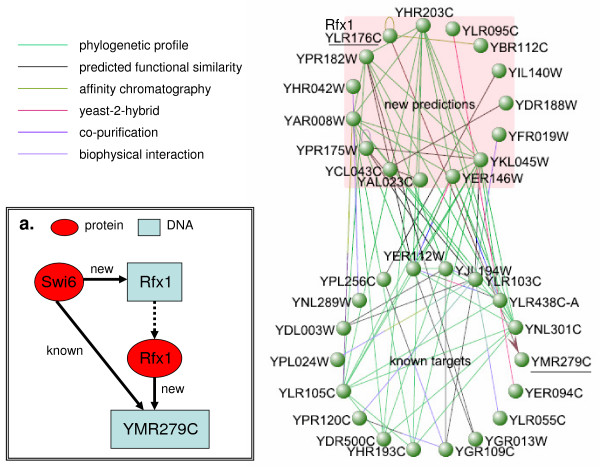
**Partial Regulatory Network of Swi6**. A sub-network showing some of the new predictions for Swi6 and how they are interconnected to previously known targets, using public datasets in the VisAnt browser. Rfx1 and YMR279C are underlined in the network. The types of data used in network construction are indicated. The VisAnt toolkit [20, 21] shows links which come from many publications. Any particular type of link (*e.g*., protein-protein interaction) may represent a collection of data from several genomic datasets. Each link type is referred to in VisAnt as a "method" and each method has a unique identifier. The method IDs for the link types here include: M0037(phylogenetic profile), M0013(copurification), M0040(screened yeast-2-hybrid), M0031(other biophysical), M0046(Bayesian Predicted Interaction), M0045(affinity chromatography). Complete references and datasets are available in the VisAnt suite accessible from the website [73]. The graph is presented to show that predicted targets of Swi6 are highly interconnected to the known targets by a variety of methods, indicating possible functional relationships or physical interactions. The insert panel "a" shows and up-close schematic of the transcriptional feed-forward loop between Swi6, Rfx1, and YMR279C. Given the known functions of Rfx1, it is likely that Rfx1 represses the YMR279C gene.

Our results include a large number of subnetworks. As an example we consider known and predicted targets of the cell cycle regulator Swi6, subsets of which (18 out of 142, and 15 out of 138, respectively) are shown in Figure 2. The average cross-validated prediction accuracy in training sets is 83%. The new predictions are highly connected to known targets by a variety of the experimental and computational methods which are available for download in the VisAnt system. Both gene groups in Figure 2 contain cell cycle genes, and the most common connection is phylogenetic profiling, suggesting considerable functional similarity. The network perspective supports the prediction of common regulation of these highly interacting genes, and makes it easier to formulate testable hypotheses about the relationships of regulatory targets.

Among the new targets of Swi6 is YLR176C (Rfx1), which codes for a transcription factor that is predicted to regulate a previously known Swi6 target, YMR279C. This arrangement is a feedforward loop, suggesting that YMR279C is under combinatorial control by these two factors (Figure 2a). It should be noted that although our method independently predicts that Rfx1 is a regulator of YMR279C, Rfx1 was reported to bind the promoter of YMR279C in an early ChIP-chip study [29]. An updated analysis of those results by the same group removed YMR279C from the dataset (can be downloaded from [30]), and a subsequent ChIP-chip experiment did not show significant regulation [22]. Due to this ambiguity the Rfx1-YMR279C interaction is not in our positive set; nonetheless, it is predicted by the classifier for Rfx1.

**Figure 3:**
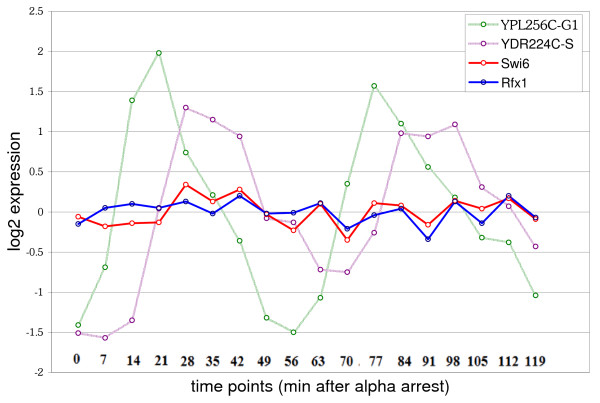
**Swi6 and Rfx1 in the Cell Cycle**. The Log2 expression values of Swi6 and a newly predicted target, Rfx1 (also a TF) during 2 cell cycles (original data from [32]). G1 and S-Phase are marked by the expression of two prototype genes, YPL256C and YDR224C, which are known to have peak expression in G1 and S-phase, respectively. Time points are indicated in minutes after alpha factor arrest. Swi6 and Rfx1 show correlated expression during the cell cycle. Time points at 21 min–42 min, and 77 min–98 min are the 8 time points mentioned in the text which show higher correlation.

Rfx1 is a repressor involved in the cell-cycle DNA damage checkpoint. Inactivation of Rfx1 occurs in response to DNA damage and causes the induction of many genes [31] (by release of repression by Rfx1). In addition to our prediction that Rfx1 is regulated by Swi6, an analysis of the expression of the two TFs during the alpha-factor arrested cell cycle time course [32] shows that their transcripts are correlated. Figure 3 shows the expression of Swi6, Rfx1, and two reference genes which show expression peaks in G1 and S-phase. Across the 18 experiments in the time course, which spans 2 cell cycles, the two factors show a correlation coefficient of 0.6.

Since Swi6 is known to be important for the G1/S transition, the expression in these specific experiments was examined more closely (noted in Figure 3). Using prototypical G1 and S phase reference genes, eight time points spanning the peak of G1 through the peak of S phase were selected. In these eight experiments Swi6 and Rfx1 show a stronger correlation of 0.73, which is statistically significant at *p *= 0.04. Interestingly, Swi6 may bind several genes in the DNA damage response pathway including Dun1 (known), Rad53 (new, *P *= 0.96), and Mec1 (potential target, *P *= 0.76). These targets are kinases known to phosphorylate and thereby activate Rfx1 in the DNA damage response pathway [31].

Finally, it has been shown that the kinase Rad53 phosphorylates Swi6, delaying progression of the cell cycle into S-phase [33]. The ultimate target of the new feed forward loop is YMR279C, which is an uncharacterized gene showing sequence similarity to membrane transport proteins. This gene shows a 9-fold induction in response to DNA damage [34,35]. Taken together, this evidence suggests that Swi6 is involved in the DNA damage response at the end of G1, and that YMR279C plays a role in DNA damage response and perhaps in cell cycle progression.

Deletion mutants of YMR279C are viable [36] but it is not known how this deletion affects the cell cycle or DNA damage response. Examining these mutants for deficiencies in growth and DNA damage response may shed some light on the true function of YMR279C. Detailed experiments including reporter assays would be needed to determine how closely interlinked Swi6, Rfx1, and YMR279C are on the transcriptional level. As a working hypothesis, it appears that Swi6 is available to activate DNA damage response genes such as Rfx1, ensuring they are present at crucial times in the cell cycle. Normally Rfx1 is repressing its targets and YMR279C will not be activated. In times of DNA damage Rfx1 is inactivated by a phosphorylation [31] allowing response genes to be induced by Swi6 and resulting in cell cycle arrest. The prediction of Swi6 binding to the Rfx1 promoter may not be of much consequence, perhaps activating Rfx1 to insure its presence during G1/S-phase. YMR279C is already known to be bound by Swi6, so the prediction that Rfx1 also binds is significant, suggesting that YMR279C will be repressed by Rfx1 until DNA damage releases it to be activated, possibly by Swi6 during the critical G1/S checkpoint.

### Prediction Analysis and Feature Rank: More on Swi6

In order to examine the regulatory implications of our model, Swi6 is again used as an example. First, the functional enrichment of Swi6 targets is examined. Swi6 interacts with Mbp1 and Swi4 during the cell cycle (G1/S transition) and in meiosis [37,38]. The known targets of Swi6 and the new predictions (throughout the range of Platt scores *P *> 0.5 → *P *> 0.95) are significantly enriched (*p *< 0.05) in the expected GO biological processes including cell cycle, regulation of cell cycle, mitotic cell cycle, DNA repair, etc. Some of the new categories for which targets at *P *> 0.95 show enrichment include chromatin assembly/disassembly (*p *= 1e-10), septin checkpoint (*p *= 2.7e-3), and lipid metabolism (*p *= 8.4e-4). These new targets fit with the current knowledge about Swi6 regulation and suggest possible new roles of action.

**Figure 4:**
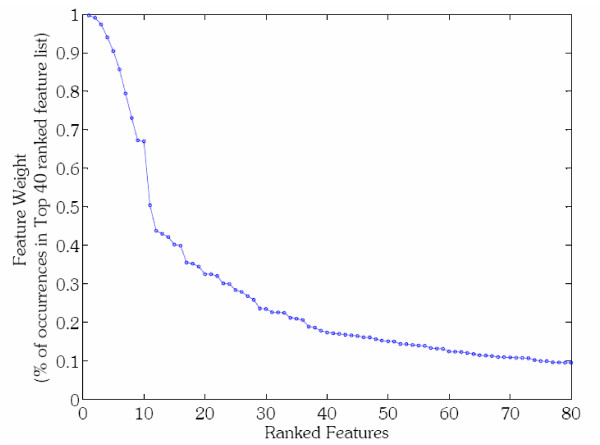
**Swi6 Feature Ranking**. A feature ranking is created on every training set during cross validation of a classifier. Since 50 classifiers are made for every TF, this results in hundreds of separate rankings (50 * #Training Points). This plot shows the frequency with which a feature shows up in the group of top 40 features. When sorted by this frequency, only the most important features remain at the top of the list. For Swi6 the first 10 features are in the top-40 of 60% of the rankings, making them consistently chosen over many resamplings of the negative set.

As described in Methods, the features for each classifier are ranked using an expanded variation of the basic SVM-RFE (**S**upport **V**ector **M**achine – **R**ecursive **F**eature **E**limination) algorithm [39]. Ranked features can be used to reveal interesting biological aspects of regulation and suggest directions for future experiments. Only a relatively small number of features show the highest discriminative ability and consistency across many subsamples of the training set (Figure 4). The first 10 features in Figure 4 have a high rank in more than 65% of the resamplings while the remaining features fall off sharply in reliability. The top ranked features for each transcription factor are available on our website (features listed in order of decreasing importance).

The most important feature for identifying known targets of Swi6 is a microarray experiment measuring expression of genes in an Mbp1 deletion mutant. This makes sense since Swi6/Mbp1 interact and function as coactivators at many promoters [37]. By *t*-test the observed expression change is significant between the negative set and the known positives (*p *= 3.7e-25), and between the negative set and the genes at *P *= 0.95 (*p *= 9.14e-27, 280 genes-known and new).

**Figure 5:**
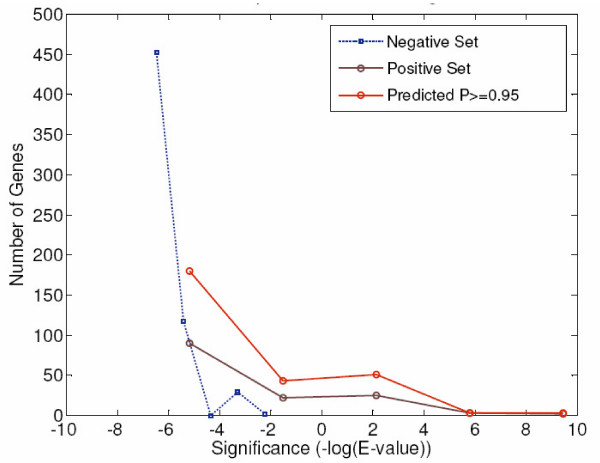
**Overrepresentation of ACGCG in Swi6 Target Promoters**. This plots the number of genes versus the E-value of ACGCG in groups of target promoters. Three categories of promoters are shown: (i) negative set promoters in blue, (ii) positive set promoters in violet, (iii) predicted targets at *P *≥ 0.95. For each category, genes are grouped into 5 equally spaced bins based on the E-value of overrepresentation of ACGCG. The center locations of those bins are plotted on the *x *axis and the number of genes in each bin is on the *y *axis. Positive and predicted target promoters of Swi6 show higher overrepresentation of ACGCG than negative set genes.

The next several highest ranked features identify the *k*-mer ACGCG/CGCGT as being important for classification by conservation and overrepresentation (features ranked 2–4, 7, and 9 indicate this *k*-mer). The *k*-mer overlaps highly with known binding sites for Swi6; for example, Swi6 binds CGCGAAA in the Cln2 promoter [24]. The overrepresentation of this sequence in the positive training set can be seen by examining the calculated *significance *values [40], which are the scores used to determine the statistical importance of each *k*-mer (see Methods). Viewing this score in the negative set genes as compared to the positives is informative, and Figure 5 plots the distribution of significance scores in the genes of the negative set, positive set, and the predicted targets at Platt scores *P *> 0.95. The graph was generated by placing the genes in each set (*i.e*., positive, negative, and predicted) into 5 equally spaced bins based on significance score. The known and predicted targets clearly show enrichment of ACGCG as compared to the negative genes.

**Figure 6:**
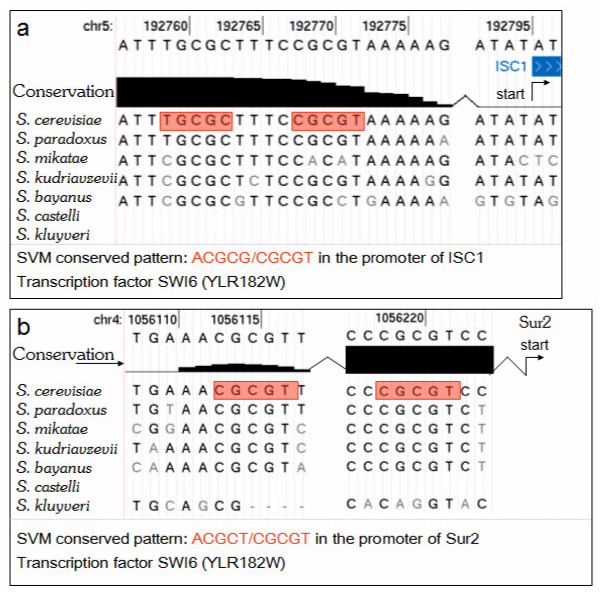
**Conservation of ACGCG in Swi6 Target Promoters**. This figure shows multi-genome alignments of selected promoter regions of Swi6 targets taken from the UCSC Genome Browser. Instances of ACGCG and its reverse complement are highlighted in red. The diagram is based on scores which are output fom the PhastCons algorithm and are pre-calculated on the UCSC Genome Browser website. The score is a posterior probability assigned to each nucleotide, giving the likelihood that the nucleotide resides in a conserved element, as defined by a hidden Markov model of "slow" and "fast" evolution. A) alignment in the promoter region of ISC1, showing two instances of the ACGCG motif. B) alignment in the promoter region of Sur2, also showing two instances of the conserved motif.

The conservation of ACGCG is ranked more highly than overrepresentation, indicating that this sequence is preserved in promoter alignments which include 7 *Saccharomyces *species. Figure 6a shows such an alignment in the promoter region of the Isc1 gene, a newly predicted target of Swi6 (Figure 6b shows a similar alignment in the Sur2 promoter). Two occurrences (highlighted in red) of the indicated *k*-mer appear in close proximity in a highly conserved segment of the Isc1 promoter. The conservation scores in Figure 6 are the output of the PhastCons algorithm [41] and are pre-calculated on the UCSC Genome Browser website (see Methods). The sequences flanking the *k*-mer in Figure 6 also show strong conservation. The SVM model does not indicate the position or which specific instances of ACGCG are conserved, but only that the *k*-mer occurs more often in conserved regions in bound promoters as opposed to non-bound promoters. This knowledge can be useful, as seen in Isc1, for narrowing down the specific sequences which may be bound.

Isc1 is an important gene since it is the only member of the sphingomyelinase (SMase) gene family present in the yeast genome. These SMases are responsible for generating ceramides, which are bioactive lipids known to modulate a variety of cellular processes including cell growth, apoptosis, and the cell cycle in yeast [42]. They also contain a newly discovered *P*-loop-like domain, which is conserved in the gene family from yeast to humans [43]. Isc1 is the closest yeast homolog to the human SMase2 gene and has thus become the model for exploring the functions of SMase genes in general.

Isc1 has been implicated in general cell growth [42], fermentative growth, and sexual reproduction [44]. Recent experiments in human and mouse models have demonstrated ceramide enhanced cancer cell death, indicating that ceramide could act synergistically with other chemotherapeutic agents [45]. Indeed, several therapeutic compounds are currently under development which modulate ceramide metabolism. The prediction that Isc1 is regulated by Swi6 (Mbp1) is interesting since it provides a direct link between cell cycle regulation and the generation of bioactive lipids via the hydrolytic pathway. Further investigation will be necessary to determine the biological validity of this link and whether the human ortholog, SMase2, shows a similar connection to the cell cycle. It is possible that ceramide production via SMase2 could serve as a target for anti-cancer therapy which can be easily studied in yeast models.

Because predicted targets of Swi6 include SMase and are enriched in genes functioning in cellular lipid metabolism, *Swi6 may have a greater role than previously appreciated in controlling lipid metabolism and its coupling to cell growth, apoptosis, and reproduction*.

### Additional Examples of Feature Ranking

**Figure 7:**
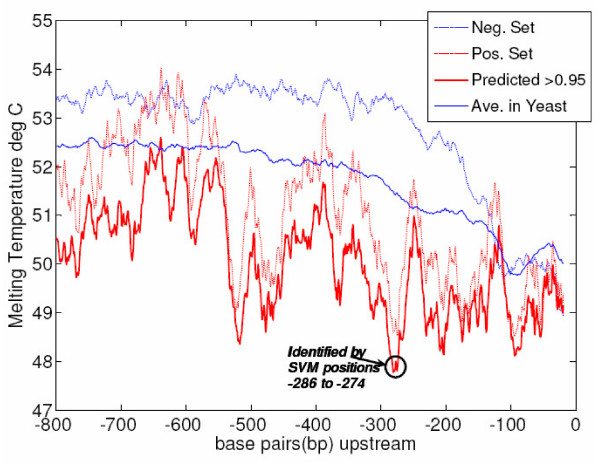
**YJL110C Target Melting Temperature Plot**. Using a 20 bp window for DNA melting temperature calculation, temperature plots are presented for the average over all 5571 yeast genes (solid blue), positive targets for YJL110C (dashed red), negatives for YJL110C (dashed blue), and at Platt score *P *≥ 0.95 (solid red). Targets of YJL110C have a lower melting temperature than the average or negative set gene. SVM feature ranking correctly identifies the window positions in which the target melting temperature is most unlike that for negative genes, suggesting altered promoter structure of the targets, which is conserved at positions -286 to -274.

As a further example of the usefulness of SVMs coupled to our feature ranking method, we briefly explore the TFs Gzf3 (YJL110C), and Ash1 (YKL185W). The feature ranking for the factor Gzf3 (YJL110C) indicates that the melting temperature at window positions -274 to -286 (see Methods) in target promoter regions is different than that in non-target promoters. Figure 7 shows a plot of the average melting temperature in sets of yeast promoters averaged within a moving 20 bp window. Known targets clearly have a reduced melting temperature at the identified positions as compared to negative set or average genes. The relationship is also present in the targets which show a Platt score > 0.95 (known and new). This group contains 72 targets, 27 of which are new predictions. Although it is unclear how the melting temperature and helix stability in this region affects regulation by Gzf3, it is possible that Gzf3 or other factors induce changes in DNA compaction or stability which alter regulation at these promoters. Binding sites of Gzf3 do not appear to be concentrated in the affected region, possibly implicating the activity of other factors at this site. In any case, feature ranking has identified specific nucleotide positions which can be tested experimentally for their role in transcriptional regulation.

The classifier for Ash1 has an average (cross validation) prediction accuracy of 88%. The most predictive feature is gene expression in She4 deletion mutants. Expression in these mutants of known targets and genes at *P *= 0.95 is significantly different than in the negative set (*p *= 5.4e^-15 ^and *p *= 6.5e^-8 ^respectively). This gene expression difference in She4 mutants is consistent with known biology since several She genes are responsible for controlling the localization, and hence appropriate regulation, of Ash1 [46-48].

### General Results and Comparison with Other Methods

As mentioned previously the cross-validation accuracy in training sets for detecting yeast binding sites (over all classifiers) is 76%. An obvious question relevant to validation is how noise in ChIP-chip data (false positives) affects assessment of the method. There are several sources of a noise. One is that the TF of interest does not contact DNA directly but interacts with a second factor which binds to the promoter. In principle this does not affect our results since the SVMs are searching for TF-promoter associations, not for specific binding sites. Other types of noise that affect allocation are more fundamental. If we consider a worst-case scenario in which the ChIP-chip targets for a regulator are near-random or too noisy, the SVM classifiers will also perform no better than random and, as we show below, our system will produce no new predictions for the TF (at the 0.95 threshold). Finally, it is important to distinguish promoter binding from transcriptional activity. Like ChIP-chip data itself, the method is determining binding rather than biological activity, the latter being inferred from a positive binding prediction. Because of the very low false positive binding rate, and because many predictions are functionally consistent with the biology of known targets we expect that the predictions made by SVM are biologically relevant.

#### Bayesian target determination

Beyer *et al*. [49] use a Bayesian framework to predict binding sites with a single unified training set of positives and negatives incorporating all TFs and targets. They use only high confidence, low-throughput binding targets as positive examples of regulation. The approach raises an interesting question regarding the trade-off between a possible disadvantage in using less prior information on the one hand, and the advantage of uniformly high quality targets on the other.

By revisiting the predictions of Beyer *et al*. [49] we find 112 TFs which are common to both their analysis and ours. There are 5156 predictions made by the Bayesian system for these factors. According to their model, which uses log-likelihood scores to assess the contributions of different data sources, 980 predictions are reported to have no ChIP-chip binding support. However, if only the ChIP-chip targets passing the *p *≤ 0.001 threshold (used by our study and [22,29]) are filtered from their total set of predictions, there are 2323 predictions made by Beyer *et al*. which were not designated as known positives by our method. In any case, ChIP-chip data was the dominant feature in their prediction system. Out of the 2323 new predictions, 1314 (56%) are also classified as positives by SVM at *P *> 0.5, while only 213 (~9%) also meet the more stringent *P *≥ 0.95 cutoff. It is not surprising that the overlap at high stringency is small considering that both methods use different types of data and completely different learning schemes. Since they are both designed to choose high-confidence targets using different datasets, they may each be predicting only a small (or different) portion of the true transcriptional regulatory network.

#### Randomized Controls

**Figure 8:**
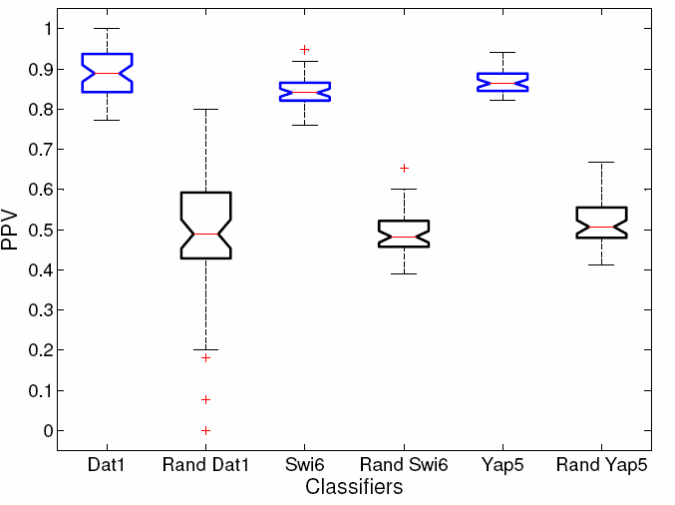
**Actual vs. Shuffled Classifiers Box-plots**. Because 50 classifiers represent each TF, cross-validation of each classifier produces a population of PPV measurements for each TF. These populations may be used to compare the significance of the actual vs. the label-shuffled classifiers (denoted with the prefix "Rand"). Here the comparison is shown for Dat1, Swi6, and Yap5 using box-and-whisker plots. The red line in each box represents a median value, and the top and bottom lines of the box represent upper and lower quartile values. If the notches on adjacent boxes do not overlap, then the population medians are considered different at 95% confidence. The whisker length is default for Matlab and is a maximum length of "1.5 times the interquartile range" [74]. The plus (+) signs represent possible outliers existing beyond this range. In each instance the real-data classifiers perform significantly better than the label-shuffled classifiers.

Here we compare TF-target classifiers to randomized data for several transcription factors. As with the actual classifiers, predictions for a randomized control must pass the 0.95 threshold across 50 separate classifiers. To illustrate the stringency implied by this requirement, the prediction procedure was implemented for three control TFs chosen according to the number of available targets (Dat1 – small, 17 targets, Yap5 – medium,72 targets, Swi6 – large, 142 targets). For each factor, the positive and negative training labels were shuffled (over all 50 random negative sets), and classifiers were built and applied to the genome. In all cases the shuffled classifiers made no predictions which passed the 0.95 threshold. Leave-one-out cross validation was also performed to determine whether the accuracy and PPV of the actual classifiers was significantly different than random. In all cases the Accuracy and PPV were significantly better than random with *p*-values less than 10^-28^. This can be seen for PPV in Figure 8, which gives box-plots of the PPVs of actual and random classifiers for the three TFs mentioned. Because these are notched-box-plots, the medians of two populations may be considered significantly different (with 95% confidence) if the notches on the boxes do not overlap.

#### Other Supervised Methods

Few other supervised approaches to target classification exist. One group [50] has applied SVMs to target detection using only gene expression data. The restriction to only gene expression limits their ability to detect interactions to cases where the TF and target exhibit correlated gene expression, which is not always the case for regulatory interactions. Like Beyer *et al*., they also create only 1 classifier to cover all TFs whereas our study makes separate classifiers for each TF. Simonis *et al*. [51] used linear discriminants to classify genes, and they employed the use of randomly selected negative sets as we do. However, their method was not used for (and wasn't intended for) predicting new targets of TFs. Instead, their purpose was only to filter out false positives from any sets of genes proposed to be co-regulated. They use only sequenced based methods whereas our procedure combines many types of heterogeneous datasets.

## Conclusion

Our SVM-based approach generates classifiers for each TF, and the significance of the predictors is assessed using cross validation and comparison to randomized control. The selection of highly enriched prediction sets is made simpler by the use of an enrichment score for each potential target gene. By incorporating many types of genome-wide measurements into a robust feature ranking system, it is possible to discover important biological aspects of regulation which are specific to each TF being studied. This has been demonstrated on the yeast cell cycle regulator, Swi6. The predicted targets of Swi6 match the known biology of the regulator and suggest possible new roles of action in bioactive lipid metabolism and the DNA damage response. Moreover, feature ranking has identified interesting biological properties of the regulator including expression change of its targets in Mbp1 deletion mutants, and over-representation/conservation of the motif ACGCG. Similar analyses can be carried out with other TFs, as shown with Gzf3 and Ash1, for which meaningful biological features are identified. Investigators may download predictions made for all TFs, view classifier accuracies, and download lists of top-ranked features for each regulator at the provided web server. Custom analyses of the full yeast transcriptional network can also be accessed online in the VisAnt browser.

Classifier accuracy is loosely correlated with the size of the positive set, where TFs with more known targets tend to have more accurate classifiers (Additional File 3). This implies that classifier performance could improve in the future as more experimental targets are discovered. The next step of this analysis is to apply these methods to the human genome and assess their reliability.

## Materials and methods

### SVM training, parameter tuning, and validation

The SVM is a statistical learning method originally developed by Vapnik [18,19,52]. SVMs are based on rigorous statistical principles and show excellent performance when making predictions on many types of large genomic datasets. The algorithm seeks a maximal separation between two groups of binary labelled (*e.g*., 0, 1 or negative, positive) training examples [53]. The training examples are feature vectors x of individual genes, each vector populated by measurements taken on genome scale datasets (see below). These measurements are the attributes of the data. A single classifier based on these features is then constructed to predict targets for each TF. Positives are genes which are known targets of the TF, and negatives are a randomly chosen subset of genes (equal in size to the positive set) which are least likely to be targets. The positives are taken from ChIP-chip experiments [23,54], Transfac 6.0 Public [24], and a list curated by Young *et al*., from which we have excluded indirect evidence such as sequence analysis and expression correlation [25]. ChIP-chip interactions of *p*-value ≤ 10^-3 ^are considered positives [54] and are not filtered using any other criteria. Since some TFs are tested under multiple experimental conditions, the negatives will be the targets with highest average *p*-values under all experiments (and they must not be targets under any condition).

The separation of targets from non-targets is accomplished by an optimization which finds a hyperplane separating the two classes [18,19]. Aside from the number of features on which to base the classifier, only one parameter must be set in our framework before training the SVM, the *C *parameter, which controls the tradeoff between the so-called margin and the classification error. Since it would be computationally expensive to choose these parameters during the training of every classifier (which would also be more likely to over-fit the data), they are first optimized on the classifier for one TF. The learned values are then applied to the remaining classifiers.

The *C *parameter adjusts the tolerance of the algorithm for misclassifications. Lower values indicate higher tolerance for errors or noisy data, with a compensating increase in the strictness of the error margin around the SVM separating hyperplane. As with feature selection (described below), the classifier for YIR018W (Yap5) was used as the prototype for parameter selection since it is known to regulate ~70 genes, which is close to the average for all TFs being analyzed. Grid selection was performed on the training set for YIR018W using many values of *C*, and classifier accuracy was measured with 5-fold cross validation. The SVM was seen to be insensitive to the choice of *C*, with most values less than 1 showing similar performance. Tested values include: [2^-7 ^2^-5 ^2^-3 ^2^-1 ^1 1.5 2 2^2 ^2^3 ^2^4 ^2^5 ^2^6^]. The value 0.0078 was chosen as this was the value reported by the SPIDER machine learning package [55] as having the best performance. In tests with other transcription factors the same value was chosen in most cases.

In previous work [17] we experimented with various varieties of SVM and found the linear SVM to be largely superior with the datasets examined here (unpublished work). By reanalyzing that original data we see that in one initial experiment where the type of SVM was allowed to vary between linear, quadratic, and cubic, the linear SVM had a higher cross-validation accuracy in 90 out of 104 cases. Similarly, using cross-validation on 104 TF classifiers, the PPV obtained by linear SVM was significantly higher by *t*-test than those using RBF (*p *= 3.62e-4) or Gaussian (*p *= 8.5e-68) SVMs. The formulas for RBF and Gaussian are quite similar and can be seen in [17]. They are used as implemented in the SPIDER machine learning toolbox [55].

Choosing negatives for TF target prediction can be difficult, since there is no defined set of genes known *not *to be targets. As in our previous work, ChIP-chip results can serve as a guide. For every TF, ChIP-chip results are used to identify genes which have the highest *p*-values (least significant) for binding under all tested conditions. From these least significant binders, the negative gene pool is chosen to be at least three times the size of the positive set, or 600 genes, whichever is larger. Recall that for each individual classifier, the number of positives and negatives is balanced. This is done by randomly under-sampling from the negative pool. Classifiers constructed on different randomly chosen negatives may give different results, since some unknown targets may be incorrectly assigned to the negative set. To smooth out these fluctuations, 50 classifiers are constructed for each transcription factor using a random resampling from the negative set. Each resampling is equal in size to the positive set and all 50 classifiers are tested using leave-one-out cross validation (LOOCV). The final performance statistics (Accuracy, PPV, etc.) are averages from the 50 trials. It is important to keep in mind that, although balanced datasets are often used in machine learning, using balanced sets means that an Accuracy of 50% is equivalent to random chance. Although many metrics exist by which to evaluate classifiers we report Accuracy and Positive Predictive Value (PPV). Accuracy is defined as the ratio of correctly classified examples to all examples classified:

$$\text{Accuracy}=\frac{\text{TP}+\text{TN}}{\text{TP}+\text{TN}+\text{FP}+\text{FN}}$$

Where TP = true positive, TN = true negative, FP = false positive, and FN = false negative. PPV is defined as the ratio of correctly predicted positives to all positive predictions:

$$\text{PPV}=\frac{\text{TP}}{\text{TP}+\text{FP}}$$

The scheme used here for classifier construction is outlined in Figure 1. To illustrate the construction and validation more concretely, a short outline is provided below. For a particular TF

1. Assemble positive set of *n *known targets. Sample *n *genes randomly from the negative pool (see above) to construct the negative set

2. Split the data for LOOCV (n-1 genes in training set, 1 gene in test set).

3. Use SVM-RFE to rank all features in the training set.

4. Construct SVM classifier on top 1500 features. Save full feature ranking.

5. Classify left out gene.

6. Repeat steps 2–5 to complete LOOCV. Save all feature rankings.

7. Calculate performance statistics (Accuracy, PPV, etc.)

8. Repeat steps 1–7 50 times.

9. Calculate final performance statistics (*i.e*., mean Accuracy, mean PPV).

A new gene can be classified by applying all 50 TF-specific classifiers to the feature vector for that gene in balanced genomic test sets. Each classification produces a Platt score (see below), and the mean of all 50 scores is used. If the mean *P *> 0.95, classify the gene as a target of the TF. The full set of feature rakings on every training set is used to calculate the final feature rank (see below).

### Genomic feature selection and ranking

The SVM algorithm can be used to select and rank data features. An important output from the algorithm is the vector **w**, which contains the learned weights of each feature. This vector points in a direction perpendicular to the hyperplane, and thus defines its orientation. Features with higher components in **w **are more useful in separating the positive and negative classes. The SVM recursive feature elimination (SVM-RFE) algorithm uses **w **to select features useful for classification [39]. The original SVM-RFE algorithm trains an SVM on a training set, and the components (attributes) of the feature vector **x **which have smallest weights are discarded [39]. The **w **vector is recalibrated and the process is repeated until the desired number of attributes remains. If feature selection were only performed one time for each TF prior to cross validation there would naturally be a risk of over-fitting, since both training and test information would be used to choose the best features. Instead we perform feature selection on each training set during cross-validation, ensuring that any over-fitting would be detected as low accuracy on the test set.

**Figure 9:**
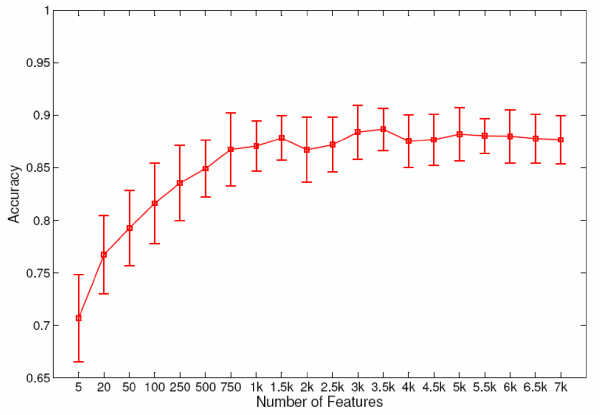
**Feature Elimination**. YIR018W (Yap5) was used as a prototype to determine the number of features needed to build a classifier. This graph shows the accuracy of classifiers for YIR018W built using different numbers of features selected using SVM-RFE. The x-axis gives the number of features selected for each tested classifier. Error-bars show one standard deviation of 50 classifiers constructed at each feature number using different sub-sampling of the negative set. 1500 features were selected as the point where accuracy reaches a plateau.

In our study, features for all genomic datasets are concatenated to produce large attribute sets for each gene. SVM-RFE is used to select relevant features during classifier training and various feature subset sizes are tested using a leave-one-out cross validation. Thus we allow the datasets to adjust, automatically selecting the most important features, irrespective of the data sets from which they originated. Once again Yap5 was used as a prototype. Figure 9 shows the effect that changes in feature number have on Yap5 classifier accuracy. Although as few as five features achieve 70% accuracy, the addition of more features continues to improve accuracy until 1500 features are selected, where accuracy is approximately 85%. 1500 is then the number chosen for the remaining TFs. The specific 1500 features are, of course, chosen individually for each TF. A more optimal procedure would be to also choose the best number of features for each classifier as this may vary from TF to TF. Such a procedure would be computationally prohibitive. In our procedure half of the features are removed during each iteration of SVM-RFE until at least 1550 features remain. Features are then removed one at a time until the target of 1500 is reached

It is important to appreciate the difference between the feature selection performed during classifier construction and the feature ranking which is later used to assess the overall usefulness of features for predicting targets of a TF. Feature selection for each classifier is simply an application of the SVM-RFE procedure. The feature rankings mentioned in the Discussion are created by compiling the individual rankings from all 50 TF classifiers. This provides a more accurate selection of features by choosing those which are ranked highly in a *majority *of training sets.

The final feature rank is achieved in the following way. After a single application of the SVM-RFE algorithm, the **w**-vector for the top 1500 features is used to determine the rank of the features in that training set, with higher weighted features having higher rank. These rankings are accumulated over every training set during cross validation of all 50 classifiers created for a TF. The result is a large set of feature rankings for a particular factor. The top 40 features (the number 40 was chosen arbitrarily) from each ranking are collected into a list, and a count is taken of the number of times each feature appears. The final rank is established by sorting the features based on the frequency of their appearance. Therefore, features which are consistently ranked high during all cross-validation trials are given a high rank. Clearly, features high on this list are reliably important for separation and robust to changes in the training set. In keeping with our example of Swi6, there are a total of 7100 feature rankings available for Swi6 features (142 examples times 50 cross validation repetitions). Figure 4 shows a plot of the features for Swi6 sorted by their occurrence in the top 40 ranked features within the 7100 rankings.

### Classifying new targets and prediction significance

As described in [27], SVMs can provide a probabilistic output which in this case measures the likelihood that any given gene is a target. Here this output is referred to simply as a Platt score or enrichment score. The intuition of this method of assigning scores is that data points which are deeper in the positive region (*i.e*., further from negative examples) are the most likely to be true positives.

Because the prior probabilities of each class (positive and negative) for a transcription factor is unknown, we choose each class to be of equal size. Thus, the Platt probabilities are strictly accurate only on the training set rather than the entire genome, where the classes would be very imbalanced. Using a balanced training set has an advantage in that some classifiers trained on balanced data often have better performance (as measured by ROC analysis) than classifiers trained on imbalanced data. This can vary, however, depending on the data sampling technique used. See [56] for an insightful discussion and comparison of sampling strategies. Thus, it should be kept in mind when evaluating the results herein that, when using balanced datasets, an Accuracy of 50% is random. As discussed above, randomized controls may be used to assess the significance of any single classifier.

Platt scores can nevertheless still be used to rank new predictions, and only those genes with a score of ≥ 0.95 are considered as potential targets in this study. The fact that our framework requires that any new target achieve an average score of ≥ 0.95 across 50 classifiers partly offers an intrinsic correction and increases the confidence in the predictions made. Randomized simulations can then be used determine whether any classifier performs significantly better than random.

Nevertheless, it may be of interest to future work to be able to correct these raw Platt scores to account for the imbalances known to exist in the full genome (where, *e.g*., 90% or more of the genes may be non-binders for any TF). As a conservative assumption about the proportion of the genome (*π*) which will be bound by a TF, take the number of genes which are bound as 10%, so *π *= 0.1. The *p*-value associated for any one gene as corrected for genomic imbalance will be given by

$$p\_full=\frac{p(1-\pi )}{p(1-2\pi )+\pi }$$

where *p *is the *p*-value (1-Platt score) in the sample and *p*_*full *is the *p*-value for the genome. As an example, if we see that a TF is predicted to bind a gene with a Platt score of 0.99, this conditional probability is equivalent to an uncorrected *p*-value of 0.01. Thus, the correction above would transform the *p*-value of 0.01 to approximately a *p*_*full *of 0.1. Note that this is a very conservative correction since it does not take into account the fact that our Platt score is the *average*over 50 classifiers.

Here and elsewhere we refer to the average, uncorrected Platt output using the upper case *P *(*e.g*., *P *> 0.99), whereas *p*-values measured by other means are shown in lower case (*e.g*., *p *< 0.01).

### Feature Datasets

Eight different types of features were used to describe genes. The first six feature sets have been used previously and their full descriptions along with many relevant references can be found in [17]. The remaining two datasets have been modified or are novel. All together these datasets comprise 15516 features. The *k*-mer based kernels are inspired by the spectrum kernel [57], the (*g*, *k*)-gappy kernel [58] and the mismatch kernel [59] which have been proposed for sequence classification. In cases were computations were made on promoter regions (datasets 1–3, 5, 7,8) the promoters were defined as the 800 base pairs upstream of the coding region (translation start cite) and S. cerevisiae promoters were downloaded from RSA tools [60]. Before analysis all features are normalized to 0 mean and standard deviation of 1. Each of the datasets is available for download at [28].

1. *k*-mers (KMER) – Feature vectors are formed by enumerating all possible strings of nucleotides of length 4, 5, and 6. The number of occurrences of each string is counted in a gene's promoter region, and this string of counts is the gene's feature vector. K-mer counting which was used in part for datasets 1, 2 and 8 were performed using code modified from a script kindly provided by Dr. William Stafford Noble of Washington University.

2. *k*-mers with Mismatch (M01) – Similar to *k*-mer counts, occurrences of all strings of length 4, 5, and 6 are counted. In addition, any string which contains only one mismatch is also considered a hit, but is given a count of 0.1 rather than 1.

3. Melting Temperature Profile (MT) – It is possible that TF binding is facilitated by conformational adjustments in promoter DNA, which depends on the stability of the helix. Some recent evidence shows correlation between sites of promoter melting, regulatory sites, and transcription initiation sites [61]. The EMBOSS [62] toolbox is used to calculate the melting temperature profiles of all yeast promoters using a sliding window of 20 bp. The feature vectors are the same as described in [17]. A temperature is calculated for each 20 bp window in the promoter, and this vector of window temperatures comprises the MT profile. 20 bp is the default window size in the EMBOSS tool.

4. Homolog Conservation (HC) – [63] BLASTP is used to compare proteins in yeast to those in 180 prokaryotic genomes. The best hit *E*-values to each genome are discretized by placing them into one of six bins using empirically determined *E*-value cut-offs. Bin numbers range from 0 (no significant hit) to 5 (very significant). Each gene then has 180 features, each for a different genome, with values ranging from 0–5, signifying the strength of the best BLASTP hit of that gene's protein to another genome.

5. *k*-mer Median Positions (Kpo) – For each possible *k*-mer (*k *= 4, 5, and 6) we record its median distance from the transcription start in each gene.

6. Expression (EXP) – Normalized log2 ratios for each gene across 1011 experiments [64] are used as features. Each gene's expression profile is normalized to a mean of 0 and standard deviation of 1. For each gene a vector 1011 long (one feature for each expression experiment) is included in the data set.

7. *k*-mer Overrepresentation (Kev) – This method counts the number of each *k*-mer appearing in a promoter and calculates the significance of its occurrence. This method is the same as that reported in our previous work [17], except that the binomial distribution is used to calculate *p*-values rather than the Poisson distribution. This is in line with the calculations described in RSA tools [40,60]. A higher Significance corresponds to a more relevant *k*-mer.

8. Conserved *k*-mers – This method for constructing a *k*-mer conservation matrix is based on output generated by the PhastCons algorithm [41,65]. PhastCons is a two state phylogenetic hidden Markov model. The underlying idea is that conserved elements evolve more slowly than non-conserved elements. Thus, it has a "slow" state for conserved DNA and a "fast" state for non-conserved, more rapidly changing sites. Given DNA sequence alignments from multiple species, PhastCons outputs a probability score for each base pair in the alignment indicating from which state the sequence arises. This probability can be interpreted as the likelihood that the base pair is part of a conserved element. Genomic alignments for seven yeast species are used to generate the probability scores, which are available for download from the USC genome browser website [66,67]. Note that the conservation scores shown in Figure 6 are from the PhastCons algorithm and were taken directly from the UCSC Genome Brower website.

During *k*-mer counting, each *k*-mer is given a unique weight depending on the average PhastCons score of its nucleotide positions. Simply weighting by the probabilities would result in missing data, since some genomic regions have no alignments. Instead we introduce a weighting scheme which increases the weight of a *k*-mer according to its conservation. Our weighting metric is:

$$\frac{1}{1-\beta P_{c}}$$

where *P*_*c *_is the average PhastCons score for a particular *k*-mer. *β *is an adjustable parameter which controls how much the conservation of a *k*-mer increases its count. In this study we choose *β *= 0.75, so that an element with a maximum conservation of 1 has a count of 4. An element which shows no conservation has the default count of 1. Increasing *β *will further emphasize the effect of conservation. This method based on PhastCons is inspired by the "marginalized motif kernel for phylogenetic shadowing" introduced in [68]. Their method uses promoter alignments and a probabilistic model of fast and slow evolution to assess conserved elements. While their method can be considered more robust when good sequence alignments are available, we adopt the approach described here so that all yeast sequences may be included in our analysis.

### Functional Analysis, Software, and Expression Data for the Swi6 Analysis

Statistical enrichment of GO biological process terms in gene sets was performed using the GO Term Finder on the Saccharomyces Genome Database website [69,70]. Most of the analysis was performed in MATLAB [71] using custom scripts along with the SPIDER software package [55].

Expression data for Swi6 was taken from [32] and their associated website [72]. Expression data for YMR279C showing cell cycle induction was from [34] and was explored using the Expression Connection [35] at the SGD website.

### VisAnt Networks

The networks (such as Figure 3) created with the VisAnt toolkit[20,21] show links which have come from many publications. Any particular type of link (*e.g*., protein-protein interaction) may represent a collection of data from several genomic datasets. Each link type is referred to in VisAnt as a "method" and each method has a unique identifier. The method IDs for the link types in this paper include: M0037(phylogenetic profile), M0013(copurification), M0040(screened yeast-2-hybrid), M0031(other biophysical), M0046(Bayesian Predicted Interaction), M0045(affinity chromatography). Complete references and datasets are available in the VisAnt suite, accessible from the VisAnt website [73].

## Competing interests

The authors declare that they have no competing interests.

## Authors' contributions

DH coded the required software in Matlab and Perl, conceived of many of the design implementations, and wrote this article. All authors made contributions to this manuscript and the experimental design, CD initially conceived and motivated this work. All authors read and approved the final manuscript.

## Reviewers' Comments

### Reviewer's report 1

Igor Jouline (Zhulin), Joint Institute for Computational Sciences, The University of Tennessee – Oak Ridge National Laboratory, Oak Ridge, TN Reviewer comments:

This study is an extension of a previously published work on machine learning for regulatory analysis and transcription factor target prediction in yeast by the same authors. Novel aspects of this work include the inclusion of new features in SVM classifiers, expansion of the transcription factor list and a case study of one of them, where some new biological insights can be gained. Overall, this is a straightforward work, which potentially can help uncovering useful biological information. Personally, I found the "principal finding #4" most interesting and appealing to a broader audience, although it is clear that it wouldn't be there without other findings reported in this study.

I do not have any major concerns. I am not impressed with the way this work is presented, perhaps because it is difficult to evaluate the true biological significance of the method. Why the Swi6 story is told in a great detail and two other transcription factors were "briefly explored"? This is out of 163 transcription factors for which the developed formalism was applied to identify their targets. Clearly, it is difficult to produce in-depth analyses for all of them, but what was the choice of the few based upon? In the absence of such explanation, one usually thinks (ignoring the presumption of innocence rule) that in the case of "the case study" biological insights were obtained, whereas in other cases it was not that impressive. Hopefully, authors can prove me (and my diabolic suspicion) wrong.

A couple of other comments: (i) I think the title of this paper is too broad and non-specific; (ii) on page 4 (last sentence) authors state that "instead of using all (available?) features to make a classifier we apply recursive feature elimination to select those that are most relevant ". It would be helpful to explain in the next sentence what those most relevant features are...

Other than that, this is certainly a strong computational study worth publishing and hopefully yeast biologists will make use of information presented here.

#### Authors' Response

Regarding the Reviewer's comment on our choice of exploring Swi6 in depth, we chose this TF because it is widely studied. It was our objective to bring practical biological insights to the publication, which is why we focused the majority of the discussion to a single factor. It was not our intention to "cherry pick" the case study, although Swi6 is one of the factors for which the method shows a higher accuracy. There are other TFs with similar accuracy measurements, though we feel that experimental validation is an important future step to corroborate these results. We would also like to direct the reader to the reviewers' comments to another manuscript wherein we apply SVM methods to predict binding sites in the human genome (Holloway *et al*. In Silico Regulatory Analysis for Exploring Human Disease Progression. 2008. Biology Direct. Pending). Comments and responses therein may also be relevant to the instant manuscript.

### Reviewer's report 2

Todd Mockler, Center for Genome Research and Biocomputing, Oregon State University

Nominated by Valerian Dolja, Department of Botany and Plant Pathology and Center for Genome Research and Biocomputing, Oregon State University

Reviewer comments:

I think this is interesting and important new work in the area of supervised learning approaches to identifying transcription factor targets. Overall, the manuscript is very well written, and the principle findings are well supported by the data presented. The methods are appropriate and sufficiently described to allow replication and/or application to other datasets or species. Additionally, the data are presented in a clear manner. I have concern about one particular figure, which should not affect publication because it doesn't affect the major conclusions relating to the figure. Figure 3 is presented as further evidence of the regulatory network connection between Swi6 and the newly predicted target Rfx1. Figure 3 is supposed to depict the association of these two genes with the cell cycle due to an apparent correlation in their expression patterns. The expression profiles of Swi6 and Rfx1 across the 18 time points have a correlation coefficient of 0.6, and a selected subset of 8 time points has a correlation coefficient of 0.73. However, unlike the G1 and S phase reference genes shown, the expression profiles of Swi6 and Rfx1 show no obvious pattern that could be associated with the cell cycle in this experiment. Moreover, the amplitudes of their changes across this time course are minimal, and could be easily mistaken for slightly correlated noise. I find this figure unimpressive, and possibly unnecessary because the major conclusions drawn from this figure are well supported by other data and cited studies as described in the manuscript.

#### Authors' Response

We thank Dr. Mockler for his comments and suggestions. We understand his reservations about Figure 3, and we agree that the expression analysis of Swi6 and Rfx1 are not required to make the manuscript complete. Nevertheless, a correlation value of 0.73 may be significant and, if nothing else, it is suggestive of further studies which may be pursued to understand the relationship between these genes.

### Reviewer's report 3

Sandor Pongor, International Centre for Genetic Engineering and Biotechnology, Trieste, Italy

Reviewer comments:

The paper is about the classification/prediction of transcription targets. It summarizes new developments to the work summarized in two preceeding publications on the same subject (Machine Learning methods for data integration, IBM J. Res. Dev., 50, pp 631–643, 2006 and "Machine learning for regulatory analysis and transcription target prediction in yeast, Systh. Synth. biol., 1: 25–46, 2007). The added value of the present manuscript is the application of recursive feature elimination for selecting relevant classifier descriptors, a technique described by Vapnik and associates (Machine Learning, 46: 389–422, 2002), a heuristic selection of attributes, and several, sometimes not entirely specified improvements to the methods, applied to a greater dataset.

While the findings are potentially interesting for a wide audience, I find the writing very technical, at times repetitive and quite difficult to follow – even for readers interested in support vector machines, string kernels and feature selection. The abstract lists four principal findings, and I am not entirely sure if and where these points are dealt with in the Results section.

For instance, the first finding reads as: "Application of the method yields an amplification of information about yeast regulators." Is "amplification of information" defined later in the text? It is then stated: "The ratio of total targets to previously known targets is greater than 2 for 11 TFs, with several having larger gains: Ash1(4), Ino2(2.6), Yaf1(2.4), and Yap6(2.4)." Apart form the general list in supplementary materials, I did not find a table in the text that would substantiate these points. The same holds for the second principal finding "Many predicted targets for TFs match well with the known biology of their regulators.". This finding should be underpinned with numerical data.

Principal finding 4 reads as follows "An analysis of global network properties highlights the transcriptional network hubs; the factors which control the most genes and the genes which are bound by the largest set of regulators. Cell-cycle and growth related regulators dominate the former; genes involved in carbon metabolism and energy generation dominate the latter." I found only one network figure which refers to a local subnetwork, but I did not find a figure or a table that would underpin this finding in a numerical way.

The authors made a large study, with many methodological improvementa that are difficult to coherently present for any audience. A better writing (removal of redundancies, a conceptual separation from previous work, and a bettter focus on added values, a clear comparison of previous and present predictions, comparison with other methods etc.) could in my opinon substantially improve this manuscript, because the work is novel and interesting. Perhaps the authors should make it clearer whether the goal is to describe methodological and Web-server details, or rather to present biological findings. It would be useful to quote examples of how a new methodology affects the efficiency of the prediction.

#### Authors' Response

Regarding Dr. Pongor's point related to "amplification of information", we intended this phrase merely to describe the fact that the method allows the prediction of many transcriptional targets, and that for some TFs the number of new predictions may be large compared to what is currently known, hence an "amplification of information". This was not intended to provide a quantitative assessment such as would be possible if metrics from Information Theory (*e.g*., Entropy measures) were employed.

Also, regarding the comments related to the network analysis, we note that global network diagrams often appear cluttered and unwieldy. For this reason we calculated basic statistics using the online VisAnt tool and chose to report the results rather than submit images which would not likely be informative.

## Supplementary Material

Additional File 1 Significant Functions of Highly Regulated Genes. This file is the output from the GO Term Finder at the Saccharomyces Genome database. Using only classifiers which had an accuracy on the training sets of ≥ 0.6 and targets identified with a Platt score *P *≥ 0.95, the genes regulated by 12 or more TFs were input into the GO Term Finder. The results show statistically enriched GO terms, *p*-values, and provide the genes annotated to those terms. Click here for file

Additional File 2 **Significant Function of Master Regulators**. This file is the output from the GO Term Finder at the Saccharomyces Genome database. Using only classifiers which had an accuracy of 0.6 and targets identified at *P *≥ 0.95, the regulators which are predicted to bind to 300 or more genes were used as input to the GO Term Finder. Click here for file

Additional File 3 **Accuracy and Number of Positives**. This figure plots the classifier accuracy on the left y-axis(blue), and the number of positives (targets) on the right y-axis(green). Classifiers are numbered on the x-axis and sorted according to increasing accuracy. A loose trend is present, showing that increasing the number of positives increases classifier accuracy. This is mainly seen when 50 or fewer positives exist. Classifiers with 20 or fewer examples tend to do poorly. Click here for file
