# Supplementary material for: Classifying transcription factor targets and discovering relevant biological features
Source: Biol Direct. 2008 May 30;3:22. doi: 10.1186/1745-6150-3-22 (PMC2441612; doi:10.1186/1745-6150-3-22)
Supplement: Additional File 1 — Significant Functions of Highly Regulated Genes. This file is the output from the GO Term Finder at the Saccharomyces Genome database. Using only classifiers which had an accuracy on the training sets of ≥ 0.6 and targets identified with a Platt score P ≥ 0.95, the genes regulated by 12 or more TFs were input into the GO Term Finder. The results show statistically enriched GO terms, p-values, and provide the genes annotated to those terms. [file 1745-6150-3-22-S1.pdf]

GOID	GO_term	Frequency	Genome Frequency	Probability	Gene(s)	Directly Annotated GOID List
722	telomerase-independent telomere maintenance	6 out of 60 genes, 10%	14 out of 7292 annotated genes, 0.1%	2.29E-09	YRF1-1 YRF1-6 YRF1-5 YRF1-2 YRF1-3 YRF1-7	722
6312	mitotic recombination	6 out of 60 genes, 10%	26 out of 7292 annotated genes, 0.3%	8.72E-08	YRF1-1 YRF1-6 YRF1-5 YRF1-2 YRF1-3 YRF1-7	722
723	telomere maintenance	7 out of 60 genes, 11.6%	180 out of 7292 annotated genes, 2.4%	0.00068	YRF1-1 YRF1-6 YRF1-5 YRF1-2 YRF1-3 YRF1-7 MOT3	722:723
6310	DNA recombination	7 out of 60 genes, 11.6%	192 out of 7292 annotated genes, 2.6%	0.00099	YRF1-1 YRF1-6 YRF1-5 YRF1-2 YER138C YRF1-3 YRF1-7	722:6319
7001	chromosome organization and biogenesis (sensu Eukaryota)	8 out of 60 genes, 13.3%	374 out of 7292 annotated genes, 5.1%	0.0113	YRF1-1 YRF1-6 YRF1-5 YRF1-2 UTH1 YRF1-3 YRF1-7 MOT3	722:1308:723
51276	chromosome organization and biogenesis	8 out of 60 genes, 13.3%	385 out of 7292 annotated genes, 5.2%	0.01328	YRF1-1 YRF1-6 YRF1-5 YRF1-2 UTH1 YRF1-3 YRF1-7 MOT3	722:1308:723
45941	positive regulation of transcription	3 out of 60 genes, 5%	69 out of 7292 annotated genes, 0.9%	0.01942	SFG1 UTH1 GAT1	45944:1308:45941
45935	positive regulation of nucleobase, nucleoside, nucleotide and nucleic acid metabolism	3 out of 60 genes, 5%	70 out of 7292 annotated genes, 0.9%	0.02016	SFG1 UTH1 GAT1	45944:1308:45941
9893	positive regulation of metabolism	3 out of 60 genes, 5%	72 out of 7292 annotated genes, 0.9%	0.02169	SFG1 UTH1 GAT1	45944:1308:45941
31325	positive regulation of cellular metabolism	3 out of 60 genes, 5%	72 out of 7292 annotated genes, 0.9%	0.02169	SFG1 UTH1 GAT1	45944:1308:45941
15980	energy derivation by oxidation of organic compounds	5 out of 60 genes,	201 out of 7292 annotated genes,	0.02483	QCR6 GID8 TSL1 ZWF1	9060:45721:5992 :9051:5977

		8.3%	2.7%		BMH1	
48522	positive regulation of cellular process	3 out of 60 genes, 5%	76 out of 7292 annotated genes, 1.0%	0.02493	SFG1 UTH1 GAT1	45944:1308:45941
51242	positive regulation of cellular physiological process	3 out of 60 genes, 5%	76 out of 7292 annotated genes, 1.0%	0.02493	SFG1 UTH1 GAT1	45944:1308:45941
43119	positive regulation of physiological process	3 out of 60 genes, 5%	76 out of 7292 annotated genes, 1.0%	0.02493	SFG1 UTH1 GAT1	45944:1308:45941
6418	tRNA aminoacylation for protein translation	2 out of 60 genes, 3.3%	30 out of 7292 annotated genes, 0.4%	0.02558	MSR1 FRS2	6420:6432
43038	amino acid activation	2 out of 60 genes, 3.3%	30 out of 7292 annotated genes, 0.4%	0.02558	MSR1 FRS2	6420:6432
43039	tRNA aminoacylation	2 out of 60 genes, 3.3%	30 out of 7292 annotated genes, 0.4%	0.02558	MSR1 FRS2	6420:6432
44262	cellular carbohydrate metabolism	5 out of 60 genes, 8.3%	204 out of 7292 annotated genes, 2.7%	0.02625	GID8 TSL1 ZWF1 EXG1 BMH1	45721:5992 :9051:6073:5977
48518	positive regulation of biological process	3 out of 60 genes, 5%	84 out of 7292 annotated genes, 1.1%	0.03216	SFG1 UTH1 GAT1	45944:1308:45941
6139	nucleobase, nucleoside, nucleotide and nucleic acid metabolism	19 out of 60 genes, 31.6%	1526 out of 7292 annotated genes, 20.9%	0.03405	YRF1-1 YRF1-6 YRF1-5 SFG1 YRF1-2 GAT2 UTH1 ZWF1 GAT1 YER138C RPA34 YRF1-3 MSR1 YRF1-7 ROX1 LSM5 HIM1 FRS2 MOT3	722:45944:6350 :1308:9051 :45941:6367:6319 :6360:6420 :122:398 :6402:6281:6432
45229	external encapsulating structure organization and biogenesis	4 out of 60 genes, 6.6%	150 out of 7292 annotated genes, 2.0%	0.03514	SED1 EXG1 CWP2 UTR2	7047

7047	cell wall organization and biogenesis	4 out of 60 genes, 6.6%	150 out of 7292 annotated genes, 2.0%	0.03514	SED1 EXG1 CWP2 UTR2	7047
6112	energy reserve metabolism	2 out of 60 genes, 3.3%	36 out of 7292 annotated genes, 0.4%	0.03571	TSL1 BMH1	5992:5977
5975	carbohydrate metabolism	5 out of 60 genes, 8.3%	223 out of 7292 annotated genes, 3.0%	0.03645	GID8 TSL1 ZWF1 EXG1 BMH1	45721:5992:9051 :6073:5977
6259	DNA metabolism	9 out of 60 genes, 15%	564 out of 7292 annotated genes, 7.7%	0.0403	YRF1-1 YRF1-6 YRF1-5 YRF1-2 UTH1 YER138C YRF1-3 YRF1-7 HIM1	722:1308 :6319:6281
6091	generation of precursor metabolites and energy	5 out of 60 genes, 8.3%	233 out of 7292 annotated genes, 3.1%	0.04269	QCR6 GID8 TSL1 ZWF1 BMH1	9060:6122:45721 :5992:9051:5977
6066	alcohol metabolism	4 out of 60 genes, 6.6%	161 out of 7292 annotated genes, 2.2%	0.04367	ERG26 GID8 ADH7 ZWF1	6696:45721 :6066:9051
6073	glucan metabolism	2 out of 60 genes, 3.3%	41 out of 7292 annotated genes, 0.5%	0.04513	EXG1 BMH1	6073:5977
7124	pseudohyphal growth	2 out of 60 genes, 3.3%	50 out of 7292 annotated genes, 0.6%	0.06408	SFG1 BMH1	7124
5976	polysaccharide metabolism	2 out of 60 genes, 3.3%	56 out of 7292 annotated genes, 0.7%	0.07794	EXG1 BMH1	6073:5977
44264	cellular polysaccharide metabolism	2 out of 60 genes, 3.3%	56 out of 7292 annotated genes, 0.7%	0.07794	EXG1 BMH1	6073:5977
45893	positive regulation of transcription, DNA-dependent	2 out of 60 genes, 3.3%	64 out of 7292 annotated genes, 0.8%	0.09772	SFG1 UTH1	45944:1308
