# Supplementary material for: Classifying transcription factor targets and discovering relevant biological features
Source: Biol Direct. 2008 May 30;3:22. doi: 10.1186/1745-6150-3-22 (PMC2441612; doi:10.1186/1745-6150-3-22)
Supplement: Additional File 2 — Significant Function of Master Regulators. This file is the output from the GO Term Finder at the Saccharomyces Genome database. Using only classifiers which had an accuracy of 0.6 and targets identified at P ≥ 0.95, the regulators which are predicted to bind to 300 or more genes were used as input to the GO Term Finder. [file 1745-6150-3-22-S2.pdf]

GOID	GO_term	Frequency	Genome Frequency	Probability	Gene(s)	Directly Annotated GOID List
6350	transcription	11 out of 13 genes, 84.6%	493 out of 7292 annotated genes, 6.7%	9.25E-12	ABF1 RCS1 SKN7 NRG1 MSN4 STE12 YAP6 UME6 CIN5 REB1 SWI4	45944:30466 :122:6350:6357 :6355:7329 :7068:6363
6355	regulation of transcription, DNA-dependent	9 out of 13 genes, 69.2%	311 out of 7292 annotated genes, 4.2%	2.85E-10	ABF1 RCS1 NRG1 MSN4 STE12 YAP6 UME6 CIN5 REB1	45944:30466:122:6357 :6355:7329:7068
45449	regulation of transcription	9 out of 13 genes, 69.2%	331 out of 7292 annotated genes, 4.5%	4.95E-10	ABF1 RCS1 NRG1 MSN4 STE12 YAP6 UME6 CIN5 REB1	45944:30466:122 :6357:6355:7329:7068
19219	regulation of nucleobase, nucleoside, nucleotide and nucleic acid metabolism	9 out of 13 genes, 69.2%	362 out of 7292 annotated genes, 4.9%	1.09E-09	ABF1 RCS1 NRG1 MSN4 STE12 YAP6 UME6 CIN5 REB1	45944:30466:122 :6357:6355:7329:7068
6139	nucleobase, nucleoside, nucleotide and nucleic acid metabolism	13 out of 13 genes, 100%	1526 out of 7292 annotated genes, 20.9%	1.47E-09	ABF1 RCS1 CBF1 MBP1 SKN7 NRG1 MSN4 STE12 YAP6 UME6 CIN5 REB1 SWI4	45944:30466:715:6260 :6338:122:6333: 6350:6357:6355 :7329:7068:7131:6363
31323	regulation of cellular metabolism	9 out of 13 genes, 69.2%	419 out of 7292 annotated genes, 5.7%	3.94E-09	ABF1 RCS1 NRG1 MSN4 STE12 YAP6 UME6 CIN5 REB1	45944:30466:122 :6357:6355:7329:7068
51244	regulation of cellular physiological process	10 out of 13 genes, 76.9%	613 out of 7292 annotated genes, 8.4%	3.97E-09	ABF1 RCS1 MBP1 NRG1 MSN4 STE12 YAP6 UME6 CIN5 REB1	45944:30466:122:74 :6357:6355:7329:7068
50794	regulation of cellular process	10 out of 13 genes, 76.9%	614 out of 7292 annotated genes, 8.4%	4.03E-09	ABF1 RCS1 MBP1 NRG1 MSN4 STE12 YAP6 UME6 CIN5 REB1	45944:30466:122:74 :6357:6355:7329:7068
50791	regulation of physiological process	10 out of 13 genes, 76.9%	631 out of 7292 annotated genes, 8.6%	5.26E-09	ABF1 RCS1 MBP1 NRG1 MSN4 STE12 YAP6 UME6 CIN5 REB1	45944:30466:122:74 :6357:6355:7329:7068
50789	regulation of biological	10 out of 13 genes, 76.9%	641 out of 7292 annotated genes,	6.13E-09	ABF1 RCS1 MBP1 NRG1 MSN4 STE12	45944:30466:122:74 :6357:6355:7329:7068

	process		8.7%		YAP6 UME6 CIN5 REB1	
19222	regulation of metabolism	9 out of 13 genes, 69.2%	445 out of 7292 annotated genes, 6.1%	6.69E-09	ABF1 RCS1 NRG1 MSN4 STE12 YAP6 UME6 CIN5 REB1	45944:30466:122 :6357:6355:7329:7068
6351	transcription, DNA-dependent	9 out of 13 genes, 69.2%	450 out of 7292 annotated genes, 6.1%	7.38E-09	ABF1 RCS1 NRG1 MSN4 STE12 YAP6 UME6 CIN5 REB1	45944:30466:122 :6357:6355:7329 :7068:6363
6357	regulation of transcription from RNA polymerase II promoter	7 out of 13 genes, 53.8%	187 out of 7292 annotated genes, 2.5%	1.09E-08	ABF1 RCS1 NRG1 STE12 YAP6 CIN5 REB1	45944:122 :6357:7329
6366	transcription from RNA polymerase II promoter	7 out of 13 genes, 53.8%	285 out of 7292 annotated genes, 3.9%	1.94E-07	ABF1 RCS1 NRG1 STE12 YAP6 CIN5 REB1	45944:122 :6357:7329
45944	positive regulation of transcription from RNA polymerase II promoter	4 out of 13 genes, 30.7%	53 out of 7292 annotated genes, 0.7%	1.89E-06	ABF1 RCS1 STE12 YAP6	45944:7329
45893	positive regulation of transcription, DNA-dependent	4 out of 13 genes, 30.7%	64 out of 7292 annotated genes, 0.8%	3.98E-06	ABF1 RCS1 STE12 YAP6	45944:7329
45941	positive regulation of transcription	4 out of 13 genes, 30.7%	69 out of 7292 annotated genes, 0.9%	5.35E-06	ABF1 RCS1 STE12 YAP6	45944:7329
45935	positive regulation of nucleobase, nucleoside, nucleotide and nucleic acid metabolism	4 out of 13 genes, 30.7%	70 out of 7292 annotated genes, 0.9%	5.66E-06	ABF1 RCS1 STE12 YAP6	45944:7329
9893	positive regulation of	4 out of 13 genes, 30.7%	72 out of 7292 annotated genes,	6.32E-06	ABF1 RCS1 STE12 YAP6	45944:7329

	metabolism		0.9%			
31325	positive regulation of cellular metabolism	4 out of 13 genes, 30.7%	72 out of 7292 annotated genes, 0.9%	6.32E-06	ABF1 RCS1 STE12 YAP6	45944:7329
43119	positive regulation of physiological process	4 out of 13 genes, 30.7%	76 out of 7292 annotated genes, 1.0%	7.82E-06	ABF1 RCS1 STE12 YAP6	45944:7329
48522	positive regulation of cellular process	4 out of 13 genes, 30.7%	76 out of 7292 annotated genes, 1.0%	7.82E-06	ABF1 RCS1 STE12 YAP6	45944:7329
51242	positive regulation of cellular physiological process	4 out of 13 genes, 30.7%	76 out of 7292 annotated genes, 1.0%	7.82E-06	ABF1 RCS1 STE12 YAP6	45944:7329
48518	positive regulation of biological process	4 out of 13 genes, 30.7%	84 out of 7292 annotated genes, 1.1%	1.15E-05	ABF1 RCS1 STE12 YAP6	45944:7329
44238	primary metabolism	13 out of 13 genes, 100%	3206 out of 7292 annotated genes, 43.9%	2.29E-05	ABF1 RCS1 CBF1 MBP1 SKN7 NRG1 MSN4 STE12 YAP6 UME6 CIN5 REB1 SWI4	45944:30466:715 :6260:6338:122 :9086:6333:6350 :6357:6006:6355 :7329:7068:7131:6363
44237	cellular metabolism	13 out of 13 genes, 100%	3400 out of 7292 annotated genes, 46.6%	4.92E-05	ABF1 RCS1 CBF1 MBP1 SKN7 NRG1 MSN4 STE12 YAP6 UME6 CIN5 REB1 SWI4	45944:30466:715 :6260:6338:122:9086 :6333:6350: 6979:6357:6006 :1324:6355:7329 :7068:7131:6363
8152	metabolism	13 out of 13 genes, 100%	3490 out of 7292 annotated genes, 47.8%	6.91E-05	ABF1 RCS1 CBF1 MBP1 SKN7 NRG1 MSN4 STE12 YAP6 UME6 CIN5 REB1 SWI4	45944:30466:715:6260 :6338:122:9086:6333:6350 :6979:6357:6006:1324 :6355:7329 :7068:7131:6363
9628	response to	5 out of 13	314 out of 7292	0.00014	SKN7 NRG1 MSN4	6979:6970:9268

	abiotic stimulus	genes, 38.4%	annotated genes, 4.3%		STE12 CIN5	:1324:7329 :9651:42493
50896	response to stimulus	6 out of 13 genes, 46.1%	588 out of 7292 annotated genes, 8.0%	0.00028	ABF1 SKN7 NRG1 MSN4 STE12 CIN5	715:6979:6970:9268:1324 :6950:7329:9651:42493
42221	response to chemical stimulus	4 out of 13 genes, 30.7%	233 out of 7292 annotated genes, 3.1%	0.00059	SKN7 MSN4 STE12 CIN5	6979:1324:7329:42493
1403	invasive growth (sensu Saccharomyces)	2 out of 13 genes, 15.3%	34 out of 7292 annotated genes, 0.4%	0.00163	NRG1 STE12	1403
50875	cellular physiological process	13 out of 13 genes, 100%	4722 out of 7292 annotated genes, 64.7%	0.00352	ABF1 RCS1 CBF1 MBP1 SKN7 NRG1 MSN4 STE12 YAP6 UME6 CIN5 REB1 SWI4	45944:30466:715 :6260:6338:122:6827 :9086:6333:7059:74 :6350:6979:6357:6006 :1302:1324:6355 :7329:747:30437 :7001:45836:7068 :7131:6363:7049:82
9987	cellular process	13 out of 13 genes, 100%	4761 out of 7292 annotated genes, 65.2%	0.00391	ABF1 RCS1 CBF1 MBP1 SKN7 NRG1 MSN4 STE12 YAP6 UME6 CIN5 REB1 SWI4	45944:30466:715 :6260:6338:122:6827 :9086:6333:7059 :74:6350:6979:6357:6006 :1302:1324:6355 :7329:747:30437 :7001:45836:7068 :7131:6363:7049:82
6979	response to oxidative stress	2 out of 13 genes, 15.3%	54 out of 7292 annotated genes, 0.7%	0.00405	SKN7 MSN4	6979:1324
7582	physiological process	13 out of 13 genes, 100%	4790 out of 7292 annotated genes, 65.6%	0.00423	ABF1 RCS1 CBF1 MBP1 SKN7 NRG1 MSN4 STE12 YAP6 UME6 CIN5 REB1 SWI4	45944:30466:715 :6260:6338:122:6827 :9086:6333:7059:74 :6350:6979:6357:6006 :1302:1324:6355 :7329:747:30437 :7001:45836:7068 :7131:6363:7049:82

6800	oxygen and reactive oxygen species metabolism	2 out of 13 genes, 15.3%	56 out of 7292 annotated genes, 0.7%	0.00434	SKN7 MSN4	6979:1324
6970	response to osmotic stress	2 out of 13 genes, 15.3%	61 out of 7292 annotated genes, 0.8%	0.00513	SKN7 CIN5	6970:9651
6950	response to stress	4 out of 13 genes, 30.7%	426 out of 7292 annotated genes, 5.8%	0.00543	ABF1 SKN7 MSN4 CIN5	715:6979:6970 :1324:6950:9651
30447	filamentous growth	2 out of 13 genes, 15.3%	91 out of 7292 annotated genes, 1.2%	0.01108	NRG1 STE12	1403:7124
6333	chromatin assembly or disassembly	2 out of 13 genes, 15.3%	100 out of 7292 annotated genes, 1.3%	0.01326	ABF1 CBF1	30466:6333
6259	DNA metabolism	4 out of 13 genes, 30.7%	564 out of 7292 annotated genes, 7.7%	0.01449	ABF1 CBF1 MBP1 UME6	30466:715:6260 :6338:6333:7131
6260	DNA replication	2 out of 13 genes, 15.3%	105 out of 7292 annotated genes, 1.4%	0.01455	ABF1 MBP1	6260
45892	negative regulation of transcription, DNA-dependent	2 out of 13 genes, 15.3%	125 out of 7292 annotated genes, 1.7%	0.02021	ABF1 UME6	30466:122:7068
40007	growth	2 out of 13 genes, 15.3%	125 out of 7292 annotated genes, 1.7%	0.02021	NRG1 STE12	1403:7124
16481	negative regulation of transcription	2 out of 13 genes, 15.3%	129 out of 7292 annotated genes, 1.7%	0.02144	ABF1 UME6	30466:122:7068
45934	negative regulation of nucleobase, nucleoside, nucleotide and nucleic acid	2 out of 13 genes, 15.3%	139 out of 7292 annotated genes, 1.9%	0.02465	ABF1 UME6	30466:122:7068

	metabolism					
7001	chromosome organization and biogenesis (sensu Eukaryota)	3 out of 13 genes, 23.0%	374 out of 7292 annotated genes, 5.1%	0.02619	ABF1 CBF1 UME6	30466:6338:6333:7001
51276	chromosome organization and biogenesis	3 out of 13 genes, 23.0%	385 out of 7292 annotated genes, 5.2%	0.02824	ABF1 CBF1 UME6	30466:6338:6333:7001
31324	negative regulation of cellular metabolism	2 out of 13 genes, 15.3%	151 out of 7292 annotated genes, 2.0%	0.02874	ABF1 UME6	30466:122:7068
9892	negative regulation of metabolism	2 out of 13 genes, 15.3%	158 out of 7292 annotated genes, 2.1%	0.03125	ABF1 UME6	30466:122:7068
7049	cell cycle	3 out of 13 genes, 23.0%	406 out of 7292 annotated genes, 5.5%	0.03241	MBP1 UME6 SWI4	74:45836:7068 :7131:7049:82
51243	negative regulation of cellular physiological process	2 out of 13 genes, 15.3%	171 out of 7292 annotated genes, 2.3%	0.03613	ABF1 UME6	30466:122:7068
48523	negative regulation of cellular process	2 out of 13 genes, 15.3%	171 out of 7292 annotated genes, 2.3%	0.03613	ABF1 UME6	30466:122:7068
43118	negative regulation of physiological process	2 out of 13 genes, 15.3%	173 out of 7292 annotated genes, 2.3%	0.0369	ABF1 UME6	30466:122:7068
48519	negative regulation of biological process	2 out of 13 genes, 15.3%	179 out of 7292 annotated genes, 2.4%	0.03927	ABF1 UME6	30466:122:7068
50876	reproductive physiological process	2 out of 13 genes, 15.3%	197 out of 7292 annotated genes, 2.7%	0.04672	STE12 UME6	747:30437

48610	reproductive cellular physiological process	2 out of 13 genes, 15.3%	197 out of 7292 annotated genes, 2.7%	0.04672	STE12 UME6	747:30437
6325	establishment and/or maintenance of chromatin architecture	2 out of 13 genes, 15.3%	211 out of 7292 annotated genes, 2.8%	0.05285	ABF1 CBF1	30466:6338:6333
6323	DNA packaging	2 out of 13 genes, 15.3%	211 out of 7292 annotated genes, 2.8%	0.05285	ABF1 CBF1	30466:6338:6333
278	mitotic cell cycle	2 out of 13 genes, 15.3%	235 out of 7292 annotated genes, 3.2%	0.064	UME6 SWI4	7068:82
3	reproduction	2 out of 13 genes, 15.3%	263 out of 7292 annotated genes, 3.6%	0.07796	STE12 UME6	747:30437
