# Supplementary figures and images for: Classifying transcription factor targets and discovering relevant biological features
Source: Biol Direct. 2008 May 30;3:22. doi: 10.1186/1745-6150-3-22 (PMC2441612; doi:10.1186/1745-6150-3-22)

Accuracy and Positives in TF Classifiers

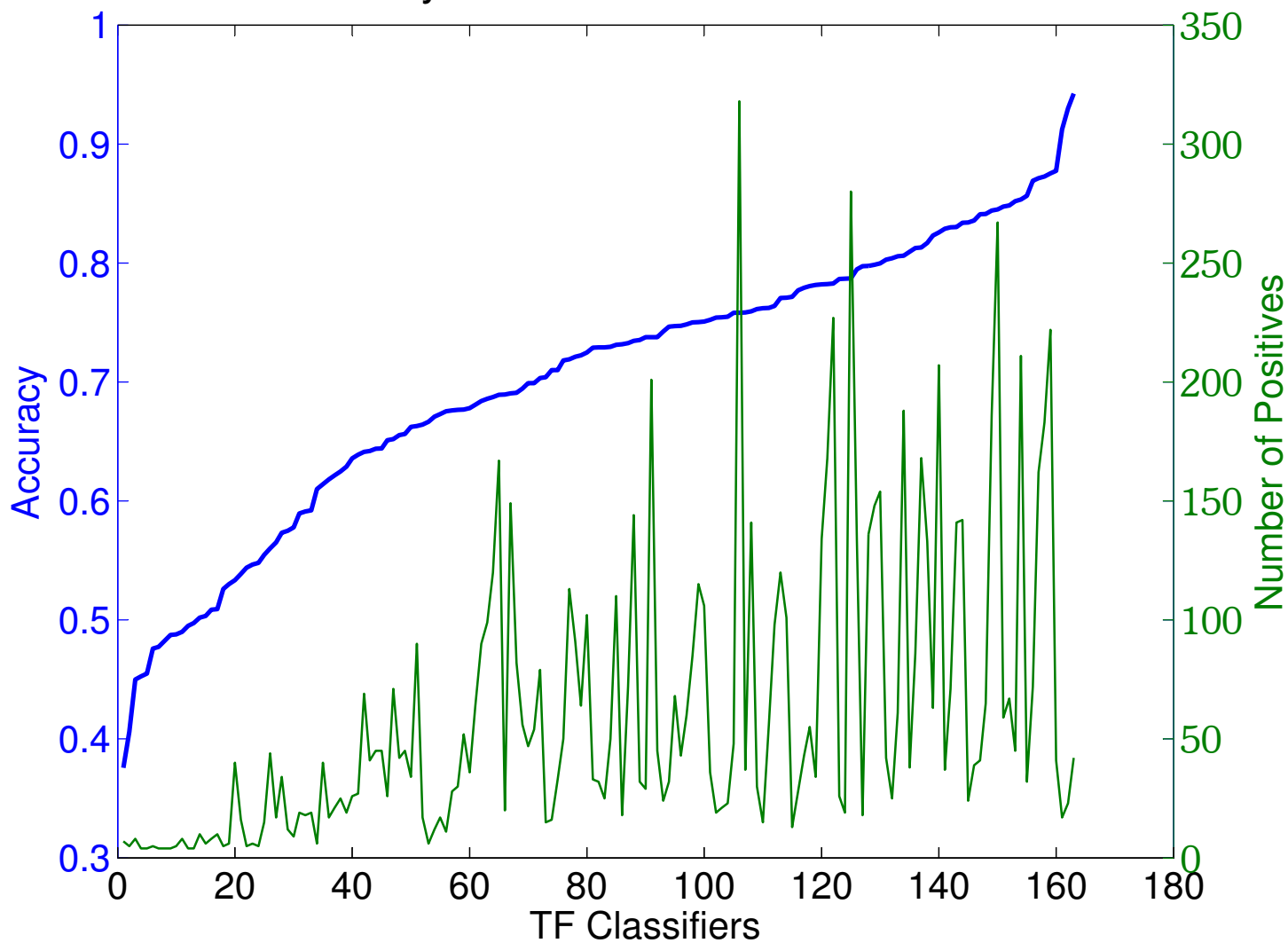

Supplement: Additional File 3 — Accuracy and Number of Positives. This figure plots the classifier accuracy on the left y-axis(blue), and the number of positives (targets) on the right y-axis(green). Classifiers are numbered on the x-axis and sorted according to increasing accuracy. A loose trend is present, showing that increasing the number of positives increases classifier accuracy. This is mainly seen when 50 or fewer positives exist. Classifiers with 20 or fewer examples tend to do poorly. [file 1745-6150-3-22-S3.pdf]
